# Inhaled Corticosteroids and Risk of *Staphylococcus aureus* Isolation in Bronchiectasis: A Register-Based Cohort Study

**DOI:** 10.3390/jcm14155207

**Published:** 2025-07-23

**Authors:** Andrea Arlund Filipsen, Karen Hougaard Frost, Josefin Eklöf, Louise Lindhardt Tønnesen, Anna Kubel Vognsen, Jonas Bredtoft Boel, Mette Pinholt, Christian Østergaard Andersen, Ram Benny Christian Dessau, Tor Biering-Sørensen, Sofie Lock Johansson, Jens-Ulrik Jensen, Pradeesh Sivapalan

**Affiliations:** 1 Copenhagen Respiratory Research, Department of Medicine, Herlev and Gentofte Hospital, University of Copenhagen, 2900 Hellerup, Denmark; 2 Department of Clinical Microbiology, Herlev and Gentofte Hospital, University of Copenhagen, 2730 Herlev, Denmark; 3 Department of Clinical Microbiology, Hvidovre University Hospital, 2650 Hvidovre, Denmark; 4 Department of Diagnostic and Infectious Disease Preparedness, Statens Serum Institut, 2300 Copenhagen, Denmark; 5 Department of Clinical Microbiology, Zealand University Hospital, 4200 Slagelse, Denmark; 6 Institute of Regional Health Research, University of Southern Denmark, 5230 Odense, Denmark; 7 Department of Cardiology, Copenhagen University Hospital, Herlev and Gentofte, 2900 Hellerup, Denmark; 8 Center for Translational Cardiology and Pragmatic Randomized Trials, Department of Biomedical Sciences, Faculty of Health and Medical Sciences, University of Copenhagen, 2200 Copenhagen, Denmark; 9 Steno Diabetes Center Copenhagen, 2730 Herlev, Denmark; 10 Section of Respiratory Medicine, Department of Medicine, Odense Universitetshospital, University of Southern Denmark, 5000 Odense, Denmark; 11 Department of Clinical Medicine, Faculty of Health Sciences, University of Copenhagen, 2200 Copenhagen, Denmark

**Keywords:** non-cystic fibrosis bronchiectasis, bronchiectasis, inhaled corticosteroids, *Staphylococcus aureus*, *S. aureus*, infection, cohort study, ICS use, cox regression, IPTW

## Abstract

**Objectives:** Non-cystic fibrosis bronchiectasis (BE) is a chronic airway disease with increasing prevalence, reduced quality of life, and increased mortality. Inhaled corticosteroids (ICS) are used in BE despite limited evidence of effect on lung function parameters. ICS may increase the risk of *Staphylococcus aureus* (*S. aureus*) infections in patients with BE, but this is unexplored. We examined the association between ICS use prior to BE diagnosis at different doses and the risk of *S. aureus* isolation in patients with BE. **Methods:** We conducted a national register-based cohort study including Danish patients with a BE diagnosis code between 2001 and 2018 with a 1-year follow-up time from the date of diagnosis. ICS exposure was categorized based on accumulated prescriptions redeemed 365 days before BE diagnosis and divided into none, low, moderate, or high use based on clinically relevant doses. A cause-specific Cox proportional hazards regression model was used to estimate the risk of *S. aureus* isolation. A sensitivity analysis, an inverse probability of treatment weighted model (IPTW), was performed. **Results:** A total of 5093 patients were included in this study. *S. aureus* was isolated in 156 patients (3.1%). High-dose ICS was associated with an increased risk of *S. aureus* isolation, HR 3.81 (95% CI 2.51; 5.79). No association for low or moderate use was found, low-dose HR 1.22 (95% CI 0.77; 1.93), and moderate-dose HR 1.24 (95% CI 0.72; 2.16). IPTW analysis yielded similar results. **Conclusions:** High-dose ICS use in patients with BE was associated with an increased risk of *S. aureus* isolation. ICS should be used cautiously in patients with BE.

## 1. Introduction

Non-cystic fibrosis bronchiectasis (BE) is a chronic airway disease characterized by persistent airway dilation, inflammation, and recurrent infections. Patients with BE often experience chronic productive cough, frequent exacerbations, and a substantial symptom burden, leading to reduced quality of life and increased length of hospitalization and risk of mortality [1,2]. The driving pathological mechanisms have previously been described as a ‘vicious vortex’ of chronic bronchial inflammation, dilation of bronchi, reduced mucociliary clearance, and chronic bacterial colonization of the airways [3]. The prevalence of BE has been increasing, with reports as high as 566/100,000, yet evidence-based treatment options remain limited [4].

Inhaled corticosteroids (ICS) are commonly prescribed in BE, despite international guidelines recommending their use only in patients with coexisting asthma or chronic obstructive pulmonary disease (COPD) [2,5]. ICS are often initiated in an attempt to reduce inflammation or exacerbation frequency, but supporting evidence in BE is sparse [6,7].

The side effects of ICS use include oral candidiasis, dysphonia, and pneumonia [8,9]. As ICS suppresses inflammation, it increases the vulnerability for respiratory infections. This has previously been demonstrated in patients with COPD for multiple different bacteria, including *Streptococcus pneumoniae* (*S. pneumoniae*), *Pseudomonas aeruginosa* (*P. aeruginosa*), and *Moraxella catarrhalis* (*M. catarrhalis*) [10,11,12], which, along with *Staphylococcus aureus* (*S. aureus*), are clinically important pathogens in BE [2]. Given the shared disruptions in airway defense mechanisms between COPD and BE, it is plausible that such effects exist in BE as well. However, there is no evidence of this.

In this national register-based cohort study, we aimed to investigate whether ICS use is associated with risk of *S. aureus* isolation among patients with BE and whether a dose–response relationship exists.

## 2. Materials and Methods

### 2.1. Data Sources

Data were obtained through the linkage of Danish national health registries using a unique personal identification number for each patient. This ensures complete follow-up. The following registers were used:

1. The Danish National Patient Register is a nationwide register that holds data on all hospital admissions since 1977 and hospital outpatient visits since 1995. The register was used to identify patients with BE and to identify comorbidities [13].
2. The Danish National Database of Reimbursed Prescriptions includes information on all reimbursed prescriptions redeemed at hospital or community pharmacies since 1995. The register was used to stratify the population based on ICS and identify other treatments and comorbidities [14].
3. Microbiological data from the Clinical Microbiology Departments in Region Zealand and the Capital Region (consisting of approximately 2.7 million inhabitants). These data were used to identify lower respiratory tract cultures positive for *S. aureus*.
4. The Danish Civil Registration System includes individual information, including unique personal identification number, sex, date of birth, and vital status [15].

### 2.2. Study Population

**Figure 1:**
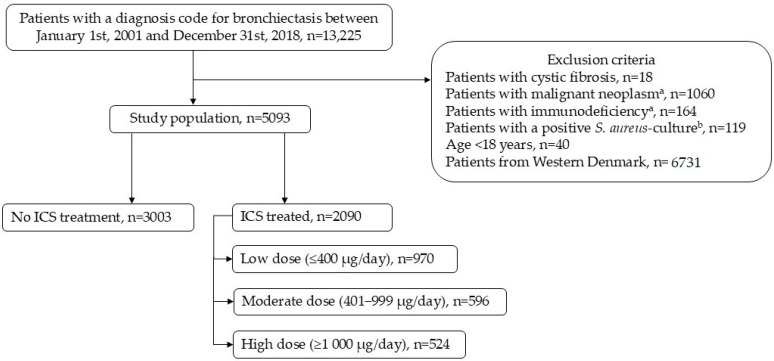
Study population: 5093 patients registered with a diagnosis code for bronchiectasis between 1 January 2001 and 31 December 2018 after inclusion and exclusion criteria. ^a^ within 5 years prior to cohort entry. ^b^ within 365 days prior to cohort entry. *S. aureus: Staphylococcus aureus*. ICS: Inhaled corticosteroids.

We conducted a national cohort study including all adults with a first-time hospital diagnosis of BE (International Classification of Diseases, 10th revision (ICD-10): DJ47) recorded in the Danish National Patient Register between 1 January 2001 and 31 December 2018. Cohort entry was defined as the date of the patients’ first BE diagnosis within these dates. We excluded patients with cystic fibrosis, a history of malignant neoplasm, or immunodeficiency within 5 years before cohort entry. Furthermore, we excluded patients with a lower respiratory tract culture positive for *S. aureus* within 365 days before cohort entry and those under the age of 18. Lastly, we excluded patients from the Danish western regions, as microbiological data from these regions were not accessible in databases (see Figure 1). This study’s power was considered adequate, given the large cohort in the eastern regions. Comorbidities were identified based on ICD-10 and Anatomical Therapeutic Chemical (ATC) codes within 5 years prior to entry into cohort. We implemented the Charlson Comorbidity Index (CCI) score as an indicator of patients’ overall physical condition using ICD-10 codes within 5 years prior to cohort entry. The calculation of the CCI score [16,17] and all ICD-10 and ATC codes used in this study are listed in Supplementary Table S1.

### 2.3. Exposure to ICS

Patients’ exposure to ICS, either as monotherapy or in fixed combination inhalers with long-acting bronchodilators, was defined as mean daily dose of all redeemed prescriptions for ICS within 365 days before their first hospital contact with a BE diagnosis code. Exposure was divided into low (≤400 µg of budesonide equivalent/day), moderate (401–999 µg of budesonide equivalent/day), and high (≥1000 µg of budesonide equivalent/day) according to NICE guidelines [18]. Non-use of ICS within 365 days prior to cohort entry was used as the reference. ICS included beclomethasone, budesonide, fluticasone, ciclesonide, and mometasone. ICS doses were converted to budesonide-equivalent doses, with beclomethasone and mometasone considered equally potent to budesonide. Beclomethasone hydrofluoroalkane and fluticasone propionate were considered twice as potent, ciclesonide 2.5 times as potent, and fluticasone furoate 10 times as potent as budesonide.

### 2.4. Outcome and Follow-Up

The primary outcome was patients’ first-time *S. aureus* isolation from a lower respiratory tract culture (first-time *S. aureus* isolation) (e.g., sputum, tracheal secretion, bronchial secretion, or bronchioalveolar lavage). The term ‘isolation’ refers to either infection or colonization with this pathogen, depending on the clinical context at the time of sampling. Samples from hospitals and general practitioners were used to identify *S. aureus* cultures on agar plates.

**Figure 2:**
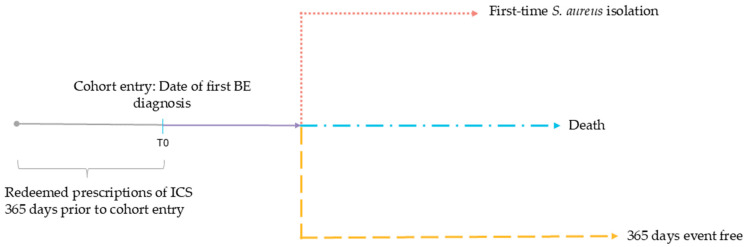
Outcome flowchart. Cohort entry between 1 January 2001 and 31 December 2018. ICS exposure was based on prescription redemptions within 365 days prior to cohort entry. Follow-up ended if the patient met the primary outcome (first-time *S. aureus* isolation), if the patient died, or if 365 days had passed after cohort entry, whichever came first. BE: non-cystic fibrosis bronchiectasis. *S. aureus*: *Staphylococcus aureus*. ICS: inhaled corticosteroids.

Follow-up ended if the patient met the primary outcome, if the patient died, or if 365 days had passed since entry into cohort (see Figure 2).

### 2.5. Statistical Analysis

Continuous and normally distributed variables were reported as mean and standard deviation (SD). Non-normal variables were reported as median and inter-quartile range (IQR). Categorical variables were reported as frequencies and proportions.

The association between ICS treatment and risk of first-time *S. aureus* isolation was estimated using a cause-specific Cox proportional hazards regression model. The model was adjusted for suspected confounders, which included age (quartiles), sex (female vs. male), CCI score (0–2, 3–4, +5), total oral corticosteroid (OCS) use (no use, low-dose OCS (<median), and high-dose OCS (≥median) within 365 days prior to cohort entry), and calendar year for entry into cohort (2001–2004, 2005–2009, 2010–2014, 2015–2018). The proportional hazards assumption was evaluated using Schoenfeld residuals, and linearity of continuous variables was tested using Martingale residuals.

To assess the effect of treatment on specific subgroups, we performed analyses investigating the interaction between ICS use and each of the following: age, sex, COPD/asthma, OCS use, and use of antibiotics 365 days prior to cohort entry.

As patients in the cohort could change exposure groups over time (e.g., from low-dose to moderate-dose group), we performed a Cox proportional hazards regression with expanded follow-up time and included ICS as a time-varying covariate. The treatment groups were categorized based on the mean daily dose of redeemed prescriptions within 90-day intervals basing the exposure group on the redeemed prescriptions in the previous interval. This method allowed for the assessment of the risk of first-time *S. aureus* isolation in the entire available person-time for all patients.

As a sensitivity analysis, an inverse probability of treatment weighted (IPTW) analysis was performed [19]. The IPTW model was implemented using multinomial propensity score weighting, based on the same variables as the adjusted main analysis. Covariate balance between treatment groups was evaluated using absolute standardized mean differences (ASMDs), with ASMD ≤ 0.1 indicating adequate balance. Subsequently, a weighted Cox proportional hazards regression model was applied to estimate the risk of detecting a positive lower airway culture for *S. aureus* in relation to ICS use.

As an additional sensitivity analysis, a Cox proportional hazards regression model was fitted, where the outcome was defined as a second positive *S. aureus* culture after an initial culture. This was performed to ensure the validity of an initial culture result.

Lastly, two sensitivity analyses exploring the risk of first-time *S. aureus* isolation from different lower respiratory tract sampling sites were performed. Here, the main outcome was defined as either a positive sputum culture or a positive lower tract culture other than sputum (e.g., tracheal secretion, bronchial secretion, or bronchoalveolar lavage). A Cox proportional hazards regression model was used to estimate the risk of *S. aureus* isolation from either sputum or lower respiratory tract cultures other than sputum.

A *p*-value of <0.05 was considered statistically significant. All analyses were performed using R statistical software (R version 4.3.3, R Core Team, Vienna, Austria). Cumulative incidence plots were produced using the R package ggsurvfit [20], and IPT weights were calculated using TWANG [21].

### 2.6. Ethics

For this study, the authors were granted access to data in nationwide registers in accordance with current Danish laws (Data Protection Agency: P-2020-1223). According to these laws, informed consent is not required for register-based studies.

### 2.7. Generative Artificial Intelligence

We used ChatGPT (OpenAI, version GPT-4o, San Francisco, CA, USA) to support language editing and phrasing. The authors reviewed and revised all content and take full responsibility for the final manuscript.

## 3. Results

We included 5093 patients, with a mean age of 62 years, and 62.0% were female. A total of 3003 patients did not receive ICS prior to BE diagnosis, while 2090 patients (41.0%) received ICS prior to BE diagnosis. Of these, 970 (19.0%) were treated with a low mean dose, 596 (11.7%) with a moderate dose, and 524 (10.3%) with a high mean dose (see Figure 1). Regarding ICS exposure in the 365 days following BE diagnosis, the number of patients in each exposure group is similar to the numbers in the year prior to diagnosis (see Supplementary Table S2).

The majority of ICS users were treated with either budesonide or fluticasone propionate, accounting for 96.3% of all ICS prescriptions. Patients in ICS treatment prior to BE diagnosis had a higher CCI score compared with those without ICS. Notably, the prevalences of COPD/asthma, heart failure, myocardial infarction, and diabetes with complications were more frequent in the ICS group (see Table 1). The median total OCS dose was 750 mg within 365 days prior to cohort entry. OCS treatment, antibiotics, and prior hospitalization within 365 days before cohort entry were also more prevalent in the group treated with ICS (see Table 1). Additionally, treatment with long-acting beta agonist (LABA) was also more prevalent in patients treated with higher doses of ICS, ranging from 15.9% in the no-ICS group to 79.2% in the high-dose group.

### 3.1. Outcome and Regression Results

Within 365 days following cohort entry, a total of 156 patients (3.1%) had *S. aureus* isolated from a lower respiratory tract culture, and 162 patients (3.2%) died (see Table 2). The median time to first-time *S. aureus* isolation was 80 days (IQR: 27–185).

When assessing treatment groups in the 90 days prior to first-time *S. aureus* isolation, 80 (51.3%) remained in the same treatment group as in the year prior to BE diagnosis. Contrary to this, 71 (45.5%) were reclassified to a lower ICS exposure group and 5 patients (3.2%) were reclassified to a higher ICS exposure group when compared with the year prior to BE diagnosis (see Supplementary Table S3).

Cox regression analysis showed an increased risk of first-time *S. aureus* isolation among patients treated with high-dose ICS prior to BE diagnosis compared with no ICS treatment (hazard ratio (HR) 3.81, 95% confidence interval (CI) 2.51; 5.79). No significantly increased risk was observed in the low-dose ICS or moderate-dose ICS group (HR 1.22, 95% CI 0.77; 1.93 and 1.24 95% CI 0.72; 2.16).

**Figure 3:**
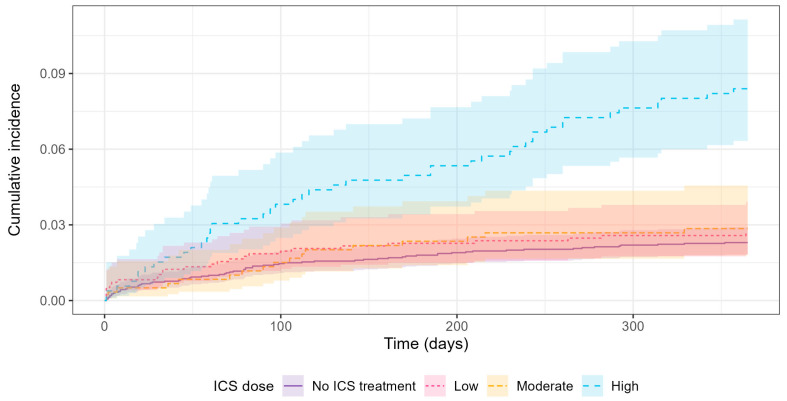
Cumulative incidence of first-time *S. aureus* isolation in patients not treated with ICS or in treatment with low-dose ICS (≤400 μg/day), moderate-dose ICS (401–999 μg/day), or high-dose ICS (≥1000 μg/day) prior to BE diagnosis. *S. aureus: Staphylococcus aureus*.

Results from the Cox regression analysis with adjusted confounders are displayed in Supplementary Table S4. The cumulative incidence of first-time *S. aureus* isolation is displayed in Figure 3. Martingale residuals for the main Cox regression analysis results showed that age as a continuous variable was not linear, and therefore, age was instead included in the regression analysis grouped in quartiles. Schoenfeld residuals showed no large breaches of the proportionality assumption.

### 3.2. Sensitivity Analyses

Interaction analyses for age, OCS use, and antibiotic use showed no interaction between subgroups. Interaction for sex was significant for high-dose ICS use prior to BE diagnosis (*p*-value = 0.046). Subgroup analysis for females receiving high-dose ICS showed an HR of 2.85 (95% CI 1.68; 4.68), and similarly, for males receiving high-dose ICS, the HR was 6.17 (95% CI 3.08; 12.30). COPD was present in 1354 patients (26.6%), asthma in 710 patients (13.9%), and other chronic pulmonary disease in 335 patients (6.6%). Among patients without a concomitant diagnosis of COPD or asthma (n = 3329), 844 (16.6%) received ICS, and 109 (2.1%) received high-dose ICS. Results for interaction analysis for COPD/asthma showed no significant interaction. Results for all interaction analyses are displayed in Supplementary Table S5–S9.

When expanding the follow-up time and allowing patients to change treatment groups over time, the crude event rates of first-time *S. aureus* isolation per person-year (py) were 3.7/py for patients not receiving ICS treatment, 4.0/py for low-dose ICS, 6.8/py for moderate-dose ICS, and 11.6/py for high-dose ICS. For death, the crude event rates were 7.9/py for patients not receiving ICS treatment, 8.7/py for low-dose ICS, 11.7/py for moderate-dose ICS, and 18.1/py for high-dose ICS. Results from the Cox regression analysis with ICS as a time-varying covariate were consistent with the main regression results and showed an HR of 1.89 (95% CI 1.44; 2.47) for high-dose ICS. Additionally, there was a slight protective effect of low-dose ICS compared with no ICS treatment. Results for the sensitivity analysis are displayed in Supplementary Table S10.

Results from IPTW analysis remained consistent with the main results, with HR for low, moderate, and high ICS dose at 1.21 (95% CI 0.74; 1.98), 1.16 (95% CI 0.65; 2.10), and 3.69 (95% CI 2.42; 5.60), respectively. After weighting, the ASMDs for all variables were below 0.1. Results are displayed in Supplementary Table S11.

In the sensitivity analysis redefining the main outcome as a second *S. aureus* isolation within 365 days of follow-up, 40 patients met the criteria. The number of deaths remained unchanged (n = 162), and Cox regression results remained similar to the main results, HR for low, moderate, and high dose at 1.47 (95% CI 0.65; 3.35), 1.70 (95% CI 0.49; 5.87), and 4.29 (95% CI 1.97; 9.33), respectively. Results are displayed in Supplementary Table S12.

Lastly, when changing the primary outcome from *S. aureus* isolated from any lower respiratory tract sample to specifically a positive sputum sample, 120 patients had a positive isolation. The regression results remained similar to the main results.

The sensitivity analysis changing the main outcome to any lower airway cultures other than sputum showed that 36 patients had a positive isolation within the 365 days of follow-up. The regression results revealed no significant effect of ICS treatment in any dose on this outcome. Results from both regression analyses are displayed in Supplementary Table S13.

## 4. Discussion

In this large national cohort study, we included more than 5000 patients with BE. We found an association between high ICS use (>1000 µg/day) prior to BE diagnosis and a more than three-fold increased hazard of first-time *S. aureus* isolation from lower airway culture compared with non-users. No significant associations were found for low and moderate ICS doses. These findings were overall consistent across sensitivity analyses, indicating a robust signal. Although a significant interaction between sexes was observed, the direction of the association remained consistent, indicating a similar overall effect. Patients receiving ICS had a higher CCI score, more frequent hospitalizations, and greater use of OCS, antibiotics, and LABA, reflecting a higher underlying disease burden.

To our knowledge, this is the first study to examine the association between ICS use and risk of first-time *S. aureus* isolation from the lower airways in a large, unselected BE population. Previous research has largely focused on other pathogens. For instance, a systematic review by Kapur et al. examined the effect of short-term (<6 months) ICS treatment on *P. aeruginosa* and *Haemophilus influenzae* (*H. influenzae*) colonization in patients with stable-state BE based on two small RCTs including in total 156 patients [6]. Neither trial found increased colonization with *P. aeruginosa* or *H. influenzae* following ICS treatment. These findings differ from ours likely due to methodological differences. First, our study focused on *S. aureus* rather than *P. aeruginosa* and *H. influenzae*, which involves distinct pathogenic mechanisms. Additionally, as the majority of cultures in our dataset were performed based on clinical indication, our study design did not support the assessment of colonization, which would require systematic and repeated sampling of included patients, as it was conducted in Kapur et al [6].

In line with this, several studies investigating the risk of infection with different microorganisms and the relation to ICS use have been performed for patients with COPD [10,11,12]. Heerfordt et al. found an increased risk of *S. pneumoniae* infection with ICS use > 1000 µg/day, and Eklöf and Johnsen found an increased risk of infection with *P. aeruginosa* and *M. catarrhalis*, respectively, with any dose of ICS. Direct comparisons between the results of these studies and our findings are difficult, as both populations and studied pathogens differ; however, several similarities are evident. COPD and BE have similar changes in airway defenses, which are dominated by neutrophilic inflammation and associated with chronic bronchial inflammation and similar lung function abnormalities [7,22], which make the populations comparable. Additionally, the method and the study period are highly aligned, further supporting the relevance of these findings in the context of our result.

### Strengths, Limitations, and Future Directions

As with any register-based study, there is a risk of misclassification bias. As exposure to ICS was based on prescription redemption within 365 days before cohort entry, some patients may have been non-adherent to treatment after cohort entry. Consequently, some patients may have been incorrectly classified as ICS users, despite non-adherence to treatment, and some may have initiated ICS use following cohort entry. Nevertheless, only a small proportion of patients changed ICS exposure groups in the 365 days before and after BE diagnosis, indicating that the majority of the patients were accurately categorized at cohort entry. Likewise, ICS use was not modeled as a time-dependent variable in the main analysis as increased risk of infection is present even months after discontinuation of treatment [23], supporting the validity of this approach. However, when evaluating the ICS exposure, among patients with first-time *S. aureus* isolation, within 90 days prior to the isolation, 71 of the 156 patients (45.5%) were reclassified from a higher to a lower ICS exposure group, indicating an extent of misclassification among these patients. This underlines the value of the sensitivity analysis including ICS as a time-dependent covariate. When using time-dependent covariates in the Cox proportional hazards regression model, there are some limitations. First, data must be cautiously structured to indicate changes in covariates over time. Done wrong, this increases the risk of misclassification bias. Second, interpretation of time-varying effects can be challenging, and if exposure time is not handled correctly, there is a risk of immortal time bias and reverse causation. In the time-dependent sensitivity analysis, results from high-dose ICS use remained consistent with the main findings, but low-dose ICS use was associated with a decreased risk of first-time *S. aureus* isolation. However, this finding should be interpreted with caution, as the result contrasts with both our main analysis, several sensitivity analyses, and the existing literature. Furthermore, the wide confidence interval and modest statistical significance reduce the robustness of the association and may reflect a chance finding rather than a true protective effect. In future research, direct measurements of medication adherence could further validate and clarify the observed associations.

In line with this, the definition of ICS exposure, using the mean daily dose within 365 days before cohort entry, was chosen to reflect two main considerations: first, to mirror real-world prescribing patterns, and second, because prescription of ICS prior to BE diagnosis may reflect treatment for another coexisting or suspected pulmonary condition, such as asthma or COPD. To account for this, an interaction analysis was performed for patients with and without concomitant COPD or asthma. This approach allowed us to address a clinically meaningful question: whether ICS use at the time of BE diagnosis, regardless of whether prescribed for pre-existing pulmonary disease or due to misclassified early BE, is associated with an increased risk of first-time *S. aureus* isolation.

The primary outcome, first-time *S. aureus* isolation from the lower respiratory tract, could indicate either colonization or infection of patients, depending on the clinical indication at the time of sampling. However, as this information was not available in databases, we were unable to distinguish between the two. Furthermore, patients with more severe illness are more likely to undergo frequent sampling, which in turn increases the likelihood of isolating *S. aureus*, introducing potential detection bias. However, sampling frequency was not included as a covariate, as it is not consistently recorded across registers. To account for this, we adjusted for disease severity using the CCI score, and care intensity was assessed using interaction analyses for the use of antibiotics and OCS.

Information on the underlying etiology of BE, which may influence the risk of *S. aureus* isolation, was not available in databases. This limitation introduces a risk of residual confounding, as the etiology of BE may influence the risk of infection. This has previously been demonstrated in patients with allogenic hemopoietic stem-cell transplantation and in post-infectious and idiopathic BE [24,25]. Future studies with access to more detailed clinical data are warranted to explore how stratification or adjustment based on BE etiology may influence microbial colonization and the risk of *S. aureus* isolation.

A key strength of this study is the use of a large well-characterized cohort of patients with BE, which enabled adjustment for numerous clinically important potential confounders. The external validity of this study is greatly increased by the size and the largely unselected population. Moreover, the robustness of the findings was supported by consistent results across multiple sensitivity analyses. To accommodate missing data on lung function, smoking history, and body mass index (BMI) in databases, the CCI score was implemented, in addition to the considerations regarding detection bias. Furthermore, we categorized patients prescribed long-acting muscarinic antagonists as diagnosed with COPD/asthma. Both strategies were applied to reduce potential residual confounding. If possible, future studies should implement suspected confounders such as lung function parameters, smoking history, BMI, and exacerbation frequency.

Although our findings support a biological plausibility of an effect of ICS, the observational nature of this study prevents any conclusions about causality between ICS use and risk of first-time *S. aureus* isolation. Nonetheless, given that we had access to and adjusted for several key confounders, it is unlikely that residual confounding alone could account for the strength of the observed HR between high-dose ICS use prior to BE diagnosis and first-time *S. aureus* isolation. Biologically, ICS may alter both innate and adaptive immune responses, as previously reported in COPD and asthma patients [26,27], potentially increasing bacterial load and shifting airway microbial composition [28].

## 5. Conclusions

This large register-based national cohort study of all patients with BE in Denmark over more than 15 years is the first to show a significantly increased risk of first-time *S. aureus* isolation in patients treated with high-dose ICS prior to BE diagnosis (HR 3.81). Our findings support a cautious approach to high-dose ICS use in BE patients, but the absolute risk remains low, and ICS should still be used when clinically indicated for other conditions. Further validation in similar cohorts is needed.

## Figures and Tables

**Table 1:** Patient characteristics at cohort entry by ICS dose in 5093 patients with BE.

	No ICS, n = 3003	Low-Dose ICS, n = 970	Moderate-Dose ICS, n = 596	High-Dose ICS, n = 524	Total, n = 5093
Age, mean (SD)	62.5 (14.4)	60.3 (14.5)	62.6 (13.3)	62.1 (13.5)	62.0 (14.2)
Female sex, n (%)	1816 (60.5)	624 (64.3)	376 (63.1)	341 (65.1)	3157 (62.0)
CCI score					
0–2, n (%)	1633 (54.4)	518 (53.4)	227 (38.1)	196 (37.4)	2574 (50.5)
3–4, n (%)	1144 (38.1)	366 (37.7)	282 (47.3)	261 (49.8)	2053 (40.3)
5+, n (%)	226 (7.5)	86 (8.9)	87 (14.6)	67 (12.8)	466 (9.1)
Heart failure, n (%)	116 (3.9)	40 (4.1)	29 (4.9)	32 (6.1)	217 (4.3)
Myocardial infarction, n (%)	53 (1.8)	20 (2.1)	12 (2.0)	21 (4.0)	106 (2.1)
Peripheral vascular disease, n (%)	95 (3.2)	26 (2.7)	13 (2.2)	23 (4.4)	157 (3.1)
Cerebrovascular disease, n (%)	120 (4.0)	30 (3.1)	24 (4.0)	16 (3.1)	190 (3.7)
Dementia, n (%)	10 (0.3)	<4	<4	<4	−
COPD/asthma, n (%)	616 (20.5)	435 (44.8)	426 (71.5)	416 (79.4)	1893 (37.2)
Connective tissue disease, n (%)	210 (7.0)	53 (5.5)	45 (7.6)	39 (7.4)	347 (6.8)
Ulcer disease, n (%)	43 (1.4)	10 (1.0)	11 (1.8)	11 (2.1)	75 (1.5)
Hemiplegia/paraplegia, n (%)	8 (0.3)	<4	<4	<4	−
Diabetes without complications, n (%)	126 (4.2)	38 (3.9)	34 (5.7)	26 (5.0)	224 (4.4)
Diabetes with chronic complications, n (%)	56 (1.9)	23 (2.4)	20 (3.4)	18 (3.4)	117 (2.3)
Mild liver disease, n (%)	32 (1.1)	5 (0.5)	6 (1.0)	7 (1.3)	50 (1.0)
Moderate/severe liver disease, n (%)	9 (0.3)	<4	<4	<4	−
Renal disease, n (%)	56 (1.9)	18 (1.9)	12 (2.0)	11 (2.1)	97 (1.9)
AIDS, n (%)	8 (0.3)	<4	<4	<4	−
OCS treatment					
No use, n (%)	2709 (90.2)	739 (76.2)	352 (59.1)	259 (49.4)	4059 (79.7)
Low dose, n (%)	179 (6.0)	153 (15.8)	136 (22.8)	119 (22.7)	587 (11.5)
High dose, n (%)	115 (3.8)	78 (8.0)	108 (18.1)	146 (27.9)	447 (8.8)
AB treatment, n (%)	1985 (66.1)	731 (75.4)	466 (78.2)	457 (87.2)	3639 (71.5)
LABA treatment, n (%)	478 (15.9)	489 (50.4)	412 (69.1)	415 (79.2)	1794 (35.2)
Hospitalization, n (%)	775 (25.8)	238 (24.5)	182 (30.5)	171 (32.6)	1366 (26.8)

Baseline characteristics of patients with BE not treated with ICS or treated with ≤400 µg/day (low-dose ICS group), 401–999 µg/day (moderate-dose ICS group), or ≥1000 µg/day (high-dose ICS group) of budesonide equivalent doses prior to BE diagnosis. Comorbidities were identified within 5 years prior to entry into cohort. All treatments and hospitalizations were identified within 365 days prior to cohort entry. Low-dose OCS ≤ 750 mg/year. High-dose OCS > 750 mg/year. ICS: inhaled corticosteroids. BE: non-cystic fibrosis bronchiectasis. SD: standard deviation. CCI: Charlson Comorbidity Index. COPD: chronic obstructive pulmonary disease. AIDS: acquired immunodeficiency syndrome. OCS: oral corticosteroid. AB: antibiotic.

**Table 2:** Outcomes within 365 days following cohort entry.

Outcome	No ICS, n = 3003	Low-Dose ICS, n = 970	Moderate-Dose ICS, n = 596	High-Dose ICS, n = 524	Total, n = 5093
First-time *S. aureus* isolation, n (%)	69 (2.3)	26 (2.7)	17 (2.9)	44 (8.4)	156 (3.1)
Death, n (%)	81 (2.7)	19 (2.0)	30 (5.0)	32 (6.1)	162 (3.2)

ICS: inhaled corticosteroids. *S. aureus*: *Staphylococcus aureus*.

## Data Availability

Restrictions apply to the availability of these data. Data were obtained from the Danish National Health Authority and are available at https://sundhedsdatastyrelsen.dk/da/forskerservice/ansog-om-data (accessed on 21 May 2025) with the permission of the Danish National Health Authority.

## Supplementary Materials

The following supporting information can be downloaded at https://www.mdpi.com/article/10.3390/jcm14155207/s1: Table S1: ICD-10 and ATC codes. Table S2: Number of patients in ICS exposure groups 365 days before and after BE diagnosis. Table S3: Numbers of patients in ICS exposure groups at cohort entry and 90 days prior to first-time *S. aureus* isolation. Table S4: Cause-specific Cox proportional hazards regression results with adjusted confounders. Table S5: Interaction analysis for sex. Table S6: Interaction analysis for age. Table S7: Interaction analysis for concomitant COPD/asthma. Table S8: Interaction analysis for OCS treatment. Table S9: Interaction analysis for antibiotic treatment. Table: S10: Results from Cox proportional hazards regression with ICS exposure as a time-varying covariate. Table S11: Results for IPTW analysis. Table S12: Sensitivity analysis for 2nd *S. aureus* isolation. Table S13: Sensitivity analysis for lower respiratory tract sampling sites.

## Abbreviations

ASMD	absolute standardized mean difference
ATC	Anatomical Therapeutic Chemical Classification System
BE	non-cystic fibrosis bronchiectasis
BMI	body mass index
CCI	Charlson Comorbidity Index
CI	confidence interval
COPD	chronic obstructive pulmonary disease
First-time *S. aureus* isolation	first-time *S. aureus* isolation from a lower respiratory tract sample
*H. influenzae*	*Haemophilus influenzae*
HR	hazard ratio
ICD-10	International Classification of Diseases and Related Health Problems 10th Revision
ICS	inhaled corticosteroids
IPTW	inverse probability of treatment weighted model
IQR	inter-quartile range
LABA	long-acting beta agonist
*M. catarrhalis*	*Moraxella catarrhalis*
OCS	oral corticosteroids
*P. aeruginosa*	*Pseudomonas aeruginosa*
Py	person-year
SD	standard deviation
*S. aureus*	*Staphylococcus aureus*
*S. pneumoniae*	*Streptococcus pneumoniae*
