# Supplementary material for: Inhaled Corticosteroids and Risk of Staphylococcus aureus Isolation in Bronchiectasis: A Register-Based Cohort Study
Source: J Clin Med. 2025 Jul 23;14(15):5207. doi: 10.3390/jcm14155207 (PMC12347263; doi:10.3390/jcm14155207)
Supplement: Supplementary file 1 [file jcm-14-05207-s001.zip › jcm-3701179-supplementary.pdf]

Supplementary Materials

Content

Supplementary Table S1 – ICD-10 and ATC codes 2

Supplementary Table S2 - Number of patients in ICS exposure groups 365 days before and after BE diagnosis 4

Supplementary Table S3 - Numbers of patients in ICS exposure groups at cohort entry and 90 days prior to first-time *S. aureus* isolation 5

Supplementary Table S4 - Cause-specific Cox proportional hazards regression results with adjusted confounders 6

Supplementary Table S5 – Interaction analysis for sex 7

Supplementary Table S6 – Interaction analysis for age 8

Supplementary Table S7 – Interaction analysis for concomitant COPD/asthma 9

Supplementary Table S8 – Interaction analysis for OCS treatment 10

Supplementary Table S9 – Interaction analysis for antibiotic treatment 11

Supplementary Table S10 – Results from Cox proportional hazards regression with ICS exposure as a time-varying covariate 12

Supplementary Table S11 – Results from IPTW analysis 13

Supplementary Table S12 – Sensitivity analysis for 2nd *S. aureus* isolation 14

Supplementary Table S13 – Sensitivity analysis for lower respiratory tract sampling sites 15

Supplementary Table S1 – ICD-10 and ATC codes

Criteria	ICD-10 or ATC code	
**Inclusion criteria**		
Diagnosis code for bronchiectasis	DJ47	
**Exclusion criteria**		
Cystic fibrosis	DE84, DQ33	
Malignant neoplasm	DC00-DC97	
Immunodeficiency	DD80-84, DD85, DD89	
Disease-modifying anti-rheumatic drugs	L04AX03, L01AA01, A07EC01, L04AD01, L04AA13, L04AX01, L04AA06, P01BA02	
**Age/Comorbidities**		**Weight for CCI score calculation**
Age 50-59		+1
Age 60-69		+2
Age 70-79		+3
Age >80		+4
Heart failure	DI50, DI11.0, DI13.0, DI13.2	+1
Myocardial infarction	DI21, DI22, DI23	+1
Peripheral vascular disease	DI70, DI71, DI72, DI73, DI74, DI77	+1
Cerebrovascular disease	DI60-69, DG45, DG46	+1
Dementia	DF00-03, DF05.1, DG30	+1
COPD/asthma	DJ40-47, DJ60-67, DJ68.4, DJ70.1, DJ70.3, DJ84.1, DJ92.0, DJ96.1, DJ98.2, DJ98.3 Or ATC R03BB, R03AL	+1
Connective tissue disease	DM05, DM06, DM08, DM09, DM30-36, DD86	+1
Ulcer disease	DK21.1, DK25-28	+1
Hemiplegia/paraplegia	DG81, DG82	+2
Diabetes mellitus	DE10.0, DE10.1, DE10.9, DE11.0, DE11.1, DE11.9	+1
Diabetes mellitus with chronic complications	DE10.2-10.8, DE11.2-11.8	+2
Mild liver disease	DB18, DK70.1, DK70.2, DK70.3, DK70.9, DK71, DK73, DK74, DK76	+1
Moderate/severe liver disease	DB15.0, DB16.0, DB16.2, DB19.0, DK70.4, DK72, DK76.6, DI85	+3
Moderate/severe renal disease	DI12, DI13, DN00-05, DN07, DN11, DN14, DN17-19, DQ61	+2
AIDS	DB21-24	+6
**Treatments**		
Antibiotics	J01CE02, J01CA04, J01CR02, J01FA01, J01FA09, J01FA06, J01FA10, J01MA02, J01MA14, J01AA02	
LABA	R03AC	

OCS	H02AB06, H02AB07

Supplementary Table S1 – ICD-10 and ATC-codes used in the study. All comorbidities are within five years before cohort entry, and all treatments are within 365 days before cohort entry. ICD-10: International Classification of Diseases, 10th edition. ATC: Anatomic Therapeutic Chemical Classification. CCI: Charlson Comorbidity Index. COPD: Chronic obstructive pulmonary disease. AIDS: Acquired Immunodeficiency Syndrome. LABA: Long-acting beta agonist. OCS: Oral corticosteroid.

**Supplementary Table S2 - Number of patients in ICS exposure groups 365 days before and after BE diagnosis**					
**ICS exposure group**	**No ICS**	**Low-dose ICS**	**Moderate-dose ICS**	**High-dose ICS**	**Total**
**Prior to BE diagnosis, n (%)**	3,003 (59.0)	970 (19.0)	596 (11.7)	524 (10.3)	5,093 (100)
**Following BE diagnosis, n (%)**	2,996 (58.8)	935 (18.4)	622 (12.2)	540 (10.6)	5,093 (100)

Supplementary Table S2 - Number of patients categorized by ICS exposure 365 days prior to and following BE diagnosis: no ICS group or stratified into a low-dose (≤400 µg/day), moderate-dose (401-999 µg/day) and high-dose (≥1,000 µg/day) ICS exposure group. ICS: inhaled corticosteroids. BE: Non-cystic fibrosis bronchiectasis

Supplementary Table S3 - Numbers of patients in ICS exposure groups at cohort entry and 90 days prior to first-time *S. aureus* isolation					
**ICS exposure group**	**No ICS**	**Low-dose ICS**	**Moderate-dose ICS**	**High-dose ICS**	**Total**
**At cohort entry, n (%)**	69 (44.2)	26 (16.7)	17 (10.9)	44 (28.2)	156 (100)
**90 days prior to first-time *S. aureus* isolation, n (%)**	91 (58.3)	43 (27.6)	20 (12.8)	2 (1.3)	156 (100)

Supplementary Table S3 - Numbers of patients categorized into ICS exposure groups at cohort entry and 90 days prior to first-time S. aureus isolation. Low-dose ICS ≤400 ug/day, moderate-dose ICS 401-999 ug/day, high-dose ICS≥1000 ug/day. ICS: Inhaled corticosteroids. S. aureus: Staphylococcus aureus.

Supplementary Table S4 - Cause-specific Cox proportional hazards regression results with adjusted confounders			
	HR	95% CI	P value
No ICS treatment	Ref	-	-
Low-dose ICS	1.22	0.77; 1.93	0.392
Moderate-dose ICS	1.24	0.72; 2.16	0.436
High-dose ICS	3.81	2.51; 5.79	<0.001
CCI score 0-2	Ref	-	-
CCI score 3-4	0.83	0.50; 1.36	0.458
CCI score +5	0.99	0.50; 1.96	0.970
Female sex	Ref	-	-
Male sex	0.85	0.61; 1.20	0.349
Year 2001-2004	Ref	-	-
Year 2005-2009	1.05	0.48; 2.34	0.896
Year 2010-2014	2.08	1.03; 4.19	0.041
Year 2015-2018	1.86	0.92; 3.74	0.084
No OCS use	Ref	-	-
Low-dose OCS	0.77	0.46; 1.30	0.327
High-dose OCS	1.17	0.72; 1.90	0.530
Age, first quartile	Ref	-	-
Age, second quartile	0.88	0.52; 1.48	0.627
Age, third quartile	1.51	0.88; 2.60	0.137
Age, fourth quartile	1.48	0.77; 2.87	0.242

Supplementary Table S4 - Results from cause-specific Cox proportional hazards regression with adjusted confounders. Low-dose ICS: ≤400 μg budesonide equivalent/day. Moderate-dose ICS: 401-999 μg budesonide equivalent/day. High-dose ICS: ≥1,000 μg budesonide equivalent/day. Low-dose OCS ≤750 mg/year. High-dose OCS >750 mg/year. HR: Hazard ratio. CI: Confidence interval. ICS: Inhaled corticosteroids. CCI: Charlson Comorbidity Index. OCS: Oral corticosteroids.

**Supplementary Table S5 – Interaction analysis for sex**		
	Female	Male
No ICS treatment	Ref.	Ref.
Low-dose ICS	Ref.	0.405
Moderate-dose ICS	Ref.	0.754
High-dose ICS	Ref.	0.046

Supplementary Table S5 – P values for interaction analysis for sex. Low-dose ICS: ≤400 µg budesonide equivalent/day. Moderate-dose ICS: 401-999 µg budesonide equivalent/day. High-dose ICS: ≥1,000 µg budesonide equivalent/day. ICS: Inhaled corticosteroid.

Supplementary Table S6 – Interaction analysis for age

	First quartile	Second quartile	Third quartile	Fourth quartile
No ICS treatment	Ref.	Ref.	Ref.	Ref.
Low-dose ICS	Ref.	0.468	0.563	0.528
Moderate-dose ICS	Ref.	0.992	0.993	0.993
High-dose ICS	Ref.	0.971	0.619	0.459

Supplementary Table 6 - P values for interaction analysis for age. Low-dose ICS: ≤400 µg budesonide equivalent/day. Moderate-dose ICS: 401-999 µg budesonide equivalent/day. High-dose ICS: ≥1,000 µg budesonide equivalent/day. ICS: Inhaled corticosteroid.

Supplementary Table S7 – Interaction analysis for concomitant COPD/asthma

	No COPD/asthma	COPD/asthma
No ICS treatment	Ref.	Ref.
Low-dose ICS	Ref.	0.886
Moderate-dose ICS	Ref.	0.597
High-dose ICS	Ref.	0.715

Supplementary Table S7 – P values for interaction analysis for concomitant COPD/asthma. Low-dose ICS: ≤400 µg budesonide equivalent/day. Moderate-dose ICS: 401-999 µg budesonide equivalent/day. High-dose ICS: ≥1,000 µg budesonide equivalent/day. ICS: Inhaled corticosteroid. COPD: Chronic obstructive pulmonary disease.

**Supplementary Table S8 – Interaction analysis for OCS treatment**			
	No OCS treatment	Low-dose OCS treatment	High-dose OCS treatment
No ICS treatment	Ref.	Ref.	Ref.
Low-dose ICS	Ref.	0.744	0.311
Moderate-dose ICS	Ref.	0.835	0.820
High-dose ICS	Ref.	0.919	0.701

Supplementary Table S8 – P values for interaction analysis for OCS treatment. Low-dose ICS: ≤400 µg budesonide equivalent/day. Moderate-dose ICS: 401-999 µg budesonide equivalent/day. High-dose ICS: ≥1,000 µg budesonide equivalent/day. Low-dose OCS ≤750 mg/year. High-dose OCS >750 mg/year. ICS: Inhaled corticosteroid. OCS: Oral corticosteroids.

Supplementary Table S9 – Interaction analysis for antibiotic treatment		
	No antibiotic treatment	Antibiotic treated
No ICS treatment	Ref.	Ref.
Low-dose ICS	Ref.	0.071
Moderate-dose ICS	Ref.	0.538
High-dose ICS	Ref.	0.837

Supplementary Table S9 - P values for interaction analysis for antibiotic treatment. Low-dose ICS: ≤400 µg budesonide equivalent/day. Moderate-dose ICS: 401-999 µg budesonide equivalent/day. High-dose ICS: ≥1,000 µg budesonide equivalent/day. ICS: Inhaled corticosteroid.

**Supplementary Table S10 – Results from Cox proportional hazards regression with ICS exposure as a time-varying covariate**			
	HR	95% CI	P value
No ICS treatment	Ref.	Ref.	Ref.
Low-dose ICS	0.53	0.30; 0.95	0.033
Moderate-dose ICS	0.94	0.69; 1.29	0.717
High dose-ICS	1.89	1.44; 2.47	<0.001

Supplementary Table S10 - Results from Cox proportional hazards regression with ICS exposure as a time-varying covariate. Low-dose ICS: ≤400 µg budesonide equivalent/day. Moderate-dose ICS: 401-999 µg budesonide equivalent/day. High-dose ICS: ≥1,000 µg budesonide equivalent/day. ICS: Inhaled corticosteroids.

Supplementary Table S11 – Results from IPTW analysis

	HR	95% CI	P value
No ICS treatment	Ref.	Ref.	Ref.
Low-dose ICS	1.21	0.74; 1.98	0.439
Moderate-dose ICS	1.16	0.65; 2.10	0.611
High dose-ICS	3.69	2.42; 5.60	<0.001

Supplementary Table S11 - Results from IPTW Cox regression model. Low-dose ICS: ≤400 µg budesonide equivalent/day. Moderate-dose ICS: 401-999 µg budesonide equivalent/day. High-dose ICS: ≥1,000 µg budesonide equivalent/day. ICS: Inhaled corticosteroids.

**Supplementary Table S12 – Sensitivity analysis for 2nd *S. aureus* isolation**			
	HR	95% CI	P value
No ICS treatment	Ref	Ref	Ref
Low-dose ICS	1.47	0.65; 3.35	0.353
Moderate-dose ICS	1.70	0.49; 5.87	0.403
High dose-ICS	4.29	1.97; 9.33	<0.001

Supplementary Table 12 - Sensitivity analysis for 2nd positive S. aureus culture adjusted for age (quartiles), sex (female vs. male), CCI score (0-2, 3-4, +5), total oral corticosteroid (OCS) use (no use, low-dose OCS (<median) and high-dose OCS (≥median) within 365 days prior to cohort entry and calendar year for entry into cohort (2001-2004, 2005-2009, 2010-2014, 2015-2018). Low-dose ICS: ≤400 µg budesonide equivalent/day. Moderate-dose ICS: 401-999 µg budesonide equivalent/day. High-dose ICS: ≥1,000 µg budesonide equivalent/day. ICS: Inhaled corticosteroids. HR: Hazard ratio. CI: Confidence interval. S. aureus: Staphylococcus aureus

**Supplementary Table S13 – Sensitivity analysis for lower respiratory tract sampling sites**			
**Sputum**			
	HR	95% CI	P value
No ICS treatment	Ref	Ref	Ref
Low-dose ICS	1.20	0.69; 2.07	0.522
Moderate-dose ICS	1.43	0.77; 2.65	0.258
High dose-ICS	4.84	3.03; 7.72	<0.001
**Other (tracheal secretion, bronchial secretion, bronchoalveolar lavage)**			
	HR	95% CI	P value
No ICS treatment	Ref	Ref	Ref
Low-dose ICS	1.31	0.57; 2.99	0.526
Moderate-dose ICS	0.83	0.24; 2.87	0.771
High dose-ICS	1.41	0.46; 4.38	0.550

Supplementary Table S13 - Sensitivity analysis for lower respiratory tract sampling sites. Low-dose ICS: ≤400 µg budesonide equivalent/day. Moderate-dose ICS: 401-999 µg budesonide equivalent/day. High-dose ICS: ≥1,000 µg budesonide equivalent/day. ICS: Inhaled corticosteroids. HR: Hazard ratio. CI: Confidence interval. S. aureus: Staphylococcus aureus.
